# GENDER INEQUALITIES IN THE DISRUPTION OF LONG-TERM LIFE SATISFACTION TRAJECTORIES DURING COVID-19

**DOI:** 10.1093/geroni/igad104.1847

**Published:** 2023-12-21

**Authors:** Dario Moreno-Agostino, Jayati Das-Munshi, George Ploubidis

**Affiliations:** UCL Centre for Longitudinal Studies and ESRC Centre for Society and Mental Health, London, England, United Kingdom; IOPPN, KCL, London, England, United Kingdom; University College London, London, England, United Kingdom

## Abstract

Evidence suggests that women’s mental health has been disproportionately impacted by the COVID-19 pandemic. Time-use differences have been hypothesised as an explanatory factor, as women generally spent more time than men on psychologically taxing activities. We used data on life satisfaction spanning 25 years (1996-2021, age 26-51) from a birth cohort representing adults born in Britain in 1970 (n=6,766). We used piecewise latent growth curve models to study gender differences in the long-term trajectories of life satisfaction and in the impact of the pandemic onset on these trajectories, and whether time-use differences partly explained that differential impact. Women had consistently higher life satisfaction levels prior to the pandemic (Δintercept,unadjusted=0.204 [95% CI: 0.073, 0.336], p=.002), and then experienced a more accelerated decline with its onset (Δquad2,unadjusted=-0.019 [-0.026, -0.011], p<.001). This gap remained stable after accounting for time use differences (Δquad2,adjusted=-0.019 [-0.028, -0.011], p<.001). Nevertheless, time spent working was positively related to life satisfaction in women but negatively in men (Δworking1-8h=0.499 [-0.168, 0.829], p= .003; Δworking>8h=0.543 [0.185, 0.902], p=.003), whereas time spent doing housework was negatively related to life satisfaction in women but positively in men (Δhousework1h=-0.723 [-0.749, -0.045], p=.027). Results were robust to adjustment for the concurrent financial situation. Our study shows gender inequalities in the impact of the pandemic on the long-term life satisfaction trajectories of adults in their 50s and suggests that time-use differences do not explain these inequalities. Further research on the mechanisms underlying the differential impact of the pandemic across genders in positive mental health is needed.

